# Effectiveness of a hospital-wide educational programme for infection control to reduce the rate of healthcare associated infections and related sepsis (ALERTS)

**DOI:** 10.1186/2047-2994-4-S1-O17

**Published:** 2015-06-16

**Authors:** S Hagel, K Ludewig, A Moeser, C Pausch, A Scherag, P Gastmeier, S Harbarth, FM Brunkhorst

**Affiliations:** 1 Center for Infectious Diseases and Infection Control, Jena University Hospital (JUH), Jena, Germany; 2 Center for Sepsis Control and Care, Jena University Hospital (JUH), Jena, Germany; 3 Institute for Medical Informatics, Statistics and Epidemiology, Leipzig, Germany; 4 Research Group Clinical Epidemiology, Center for Sepsis Control and Care, Jena University Hospital (JUH), Jena, Germany; 5 Institute of Hygiene and Environmental Medicine, University Medicine, Charité, Berlin, Germany; 6 Infection Control Programme, Geneva University Hospitals, Geneva, Switzerland; 7 Paul Martini Sepsis Research Group, Jena, Germany; 8 Center of Clinical Studies, Jena University Hospital (JUH), Jena, Germany

## Introduction

The overarching objective of this clinical trial is to demonstrate the feasibility of an institutional programme to reduce the burden of Healthcare Associated Infections (HAIs) and related sepsis of at least 20%, without targeting only specific pathogens or hospital wards.

## Methods

Prospective, quasi-experimental study covering all acute care units (27 general wards, 4 ICUs, overall 819 beds) at the Jena University Hospital. Surveillance for HAIs is performed by computerized antibiotic monitoring in patients with risk factors for HAIs (i.e. catheters, operations) on a daily basis. Following the 1^st^ surveillance period (09/2011 to 08/2012) a multifaceted, pragmatic infection control programme, aimed at proper hand hygiene and bundles for the prevention of the four most common HAIs has been implemented. Subsequently, a 2^nd^ surveillance phase (04/2013 to 08/2014) was conducted to measure the effect of the infection control programme.

## Results

During the the first surveillance period 30.631 patients were admitted to the participating departments. According to CDC definitions we identified 1,637 HAIs, resulting in an overall incidence of 5.3 %. Based on clinical evaluation, irrespective of the CDC definitions, an additional 944 HAIs were detected (overall HAI rate, 8.4 % [n =2581]). A substantial proportion of patients had HAI associated severe sepsis or septic shock (lower respiratory tract infection, n = 279 [37 %]; surgical site infection, n = 114 [25 %]; primary sepsis, n = 110 [32 %]; urinary tract infection, n = 46 [8 %]; other, n = 87 [22 %]). The analysis of the second surveillance period is pending, the results however will be presented at the congress.

## Conclusion

Our numbers reveal that a high number of HAIs are missed using CDC-definitions and therefore the magnitude of the problem might be underestimated. Furthermore, a high percentage of HAIs progress from localized infection to severe sepsis or septic shock, requiring ICU treatment.

## Disclosure of interest

None declared.

